# Bioelectrical impedance vectors analysis (BIVA) in older adults according to level of physical activity and muscle strength: a comparison of classic and specific approaches

**DOI:** 10.3389/fragi.2025.1535876

**Published:** 2025-05-27

**Authors:** Ismael Figueiredo Rabelo, Shannon Farrell, Kieran F. Reid, Vanessa Ribeiro Dos Santos, Melissa Antunes, Vitor Cabrera Batista, Andreia Bauermann-Vieira, Luís Alberto Gobbo

**Affiliations:** ^1^ Skeletal Muscle Assessment Laboratory, Department of Physical Education, School of Technology and Science, São Paulo State University (UNESP), Presidente Prudente, Brazil; ^2^ Laboratory of Exercise Physiology and Physical Performance, Boston Claude D. Pepper Older Americans Independence Center for Function Promoting Therapies, Brigham and Women’s Hospital, Harvard Medical School, Boston, MA, United States

**Keywords:** bioelectrical impedance, older adults, physical activity, muscle strength, phase angle

## Abstract

**Indroduction:**

Bioelectrical Impedance Analysis (BIA) is a widely used method to assess body composition. Traditional BIA models use predictive equations without considering individual characteristics such as age, sex, and activity level. Classic Bioelectrical Impedance Vector Analysis (BIVA) standardizes raw BIA data by height, while specific BIVA (spBIVA) normalizes by body segment areas and lengths, offering a potentially more accurate assessment. With aging populations, there is growing interest in assessing age‐related body composition changes ‐ especially sarcopenia, characterized by reduced muscle mass and function. While BIVA is promising for such assessments, limited studies compare classic and specific BIVA approaches in older adults based on physical activity and muscle strength. Thus, the objective of the study is to compare classic and specific BIVA values in older adults according to their physical activity level and muscle strength.

**Methods:**

This cross‐sectional study involved 187 community‐dwelling older adults (143 women and 44 men, ≥60 years), recruited via public advertisements. Exclusion criteria included medical conditions affecting muscle mass. The Assessments included: Anthropometry (weight, height, BMI, waist, arm and calf circumferences); BIA (resistance ‐ R, reactance ‐ Xc, and phase angle ‐ PhA), measured using a 50 kHz analyzer (classic BIVA was normalized by height and specific BIVA by segmental area/length using upper arm); physical activity (measured using the Baecke Habitual Physical Activity Questionnaire); muscle strength (measured by handgrip dynamometry, cut‐off values <27 kg for men and <16 kg for women indicated low strength). For the statistical analyses, differences in BIVA parameters were evaluated using Hotelling’s T^2^ test and Mahalanobis D distances (p < 0.05).

**Results:**

Men had significantly higher body weight, height, waist circumference, and handgrip strength (p < 0.05). Classic BIVA consistently showed higher values of R and Xc than specific BIVA (differences up to 30%). PhA was higher in men, especially due to lower resistance values. Among women, those with higher physical activity levels had significantly higher PhA, indicating better cellular health. Among men, no significant differences in PhA were observed between activity levels. For muscle strength, stronger men had higher PhA values and significant vector displacement in both models; women did not show significant differences by strength level.

**Discussion and Conclusion:**

Both classic and specific BIVA approaches identified differences in impedance parameters related to physical activity and strength. Classic BIVA tends to overestimate resistance and reactance due to height normalization, while specific BIVA, using body segments, may better reflect individual morphological characteristics.

## 1 Introduction

Bioelectrical impedance (BIA) is a well-established method for assessing body composition across various populations, utilizing predictive equations to analyze the morphological and functional components. However, traditional BIA does not typically account for factors like age, sex, physical activity levels, or fitness, using a standardized approach for all individuals. Classic bioelectrical impedance vector analysis (BIVA), as introduced by Piccoli, uses raw BIA parameters–resistance (R) and reactance (Xc) – standardized by height, considering the body as a single cylindrical entity. More recently, specific BIVA (spBIVA) has been introduced, which instead standardizes BIA values based on specific body segments (arm, waist, and calf) for potentially more accurate assessments (Buffa et al., 2013; Buffa et al., 2014; Marini et al., 2013).

With global demographic shifts and an aging population, understanding body composition in older adults has become a priority. Aging leads to notable morphological and functional changes, such as an increase in body fat mass percentage, reduced muscle mass, and lower bone density and mineral content (Wright et al., 2014), which are closely linked to decreased muscle strength and physical function.

The age-related decline in muscle mass and function, termed sarcopenia, poses risks for frailty, falls, fractures, and overall mortality. Key contributions to sarcopenia include poor nutrition status, physical inactivity, and the inflammatory process resulted from excess body fat, mainly due to the rise in proinflammatory cytokines and leptins and reduced levels of adiponectin (Baumgartner et al., 2004; Ilich et al., 2014). Conversely, regular physical activity is known to preserve muscle function and independence, reducing the risk of frailty and related complications.

Although BIA and spBIVA offer promising tools for assessing these age-related changes, research on their use in older adults, particularly comparing classic and specific BIVA methods with respect to physical activity levels, muscle strength, and gender differences, remains limited. This study aims to address this knowledge gap by comparing classic and specific BIVA measures in older adults, with a focus on how physical activity and muscle strength impact the accuracy and applicability of these methods. This could advance early detection and intervention for sarcopenia in aging populations.

To date there is currently limited research observing the new techniques for using BIA and spBIVA. Therefore, there is a need for ongoing research focusing on other populations and age ranges to confirm validity. Taking into consideration the negative aspects of morphological, physical functional and functional capacity components in an advanced aged population, the application of BIA using raw parameters and the classic or specific analysis of vectors has the potential for the early detection of sarcopenia and thus promote intervention programs. Also, although both methods derive from the same raw BIA parameters, their normalization by either height (classic) or segment area/length (specific) yields different impedance vectors. This comparison highlights the potential of specific BIVA to reflect body composition nuances, especially in clinical settings assessing sarcopenia. Thus, the purpose of this study was to compare classic and specific BIVA values of older adults based on level of physical activity and muscle strength, measurements often applied for sarcopenia detection.

## 2 Materials and methods

This cross-sectional study was conducted at the Center for Studies and Laboratory for Evaluation and Prescription of Motor Activity (CELAPAM) of the department of Physical Education at the São Paulo State University (UNESP) in Presidente Prudente–São Paulo/Brazil. This research was approved by the Research Ethics Committee of the (UNESP) (CAEE 26058114.3.0000.5402).

### 2.1 Participants

Older adults aged 60 years and older who were able to attend laboratory evaluations and provide informed consent were recruited using convenience sampling via university corporate communications, online and televised advertisements.

Individuals were excluded if they resided in a long-stay institution, clinically diagnosed with a disease or medical condition affecting the dynamics of muscle mass reduction, such as, HIV/AIDS, tuberculosis or chronic kidney disease.

### 2.2 Anthropometry

Body weight and height measurements were collected at baseline. Body weight was measured using an electronic scale (Filizola^®^ Anthropometric, São Paulo, Brazil), with a maximum capacity of 180 kg and accuracy of 0.1 kg. Height was measured using a fixed stadiometer, the Sanny^®^ Standard model (São Bernardo do Campo, SP, Brazil), with a precision of 0.1 cm and a length of 2.20 m. The values obtained for weight and height were used to calculate the body mass index (BMI, in kg/m^2^) from the ratio between weight, in kg, and height, in meters squared.

### 2.3 Bioelectrical impedance and bioelectrical impedance vector analysis (BIVA)

The BIA evaluation was performed using a four-pole BIA Analyzer TM equipment (The Nutritional Solutions, Harrisville, MI, USA), with a frequency of 50 kHz and an amperage of 400 μA, when resistance (R) and reactance (Xc) values were obtained. Subsequently the phase angle (PhA), in degrees, was calculated.

Specific BIVA (Buffa et al., 2013; Buffa et al., 2014) were calculated by multiplying R and Xc measures by the correction factor A/L (A, m^2^ = area; L = length, m). Area and length were adjusted as follows.

• A = (0.45. upper arm area +0.10. waist area +0.45. calf area);
• L = 1.1. stature.

The segments (arm, waist and calf) area, in meters, were calculated as C^2^/4π (Buffa et al., 2013), where C = circumference, in cm.

Additionally, confidence ellipses were developed to compare cellular health parameters (impedance) between different groups, established from independent variables (groups according to gender, pre and post training moments, level of physical activity and strength).

Regarding the positioning of participants during the assessment, the body position were at the supine position for 5 min prior the evaluation, with the arms separated from trunk by about 30° and legs separated by about 45°. Ag/AgCl electrodes were placed in the right hemibody, at the dorsal surfaces of the wrist and ankle. Voltage electrodes are applied at midline between the prominent bone ends on the wrist (ulna and radius) and the ankle (medical and lateral malleoli). Current electrodes were placed 5 cm distal to these positions. The sites were cleaned with alcohol.

In an attempt to minimize possible estimation errors, the participants were instructed to urinate about 30 min before the measurements were taken, refrain from ingestion of food or drink in the last 4 h, avoid the practice of vigorous physical exercises for at least 24 h, abstain from the consumption of alcohol and caffeinated beverages for at least 48 h.

### 2.4 Physical activity and muscle strength

The Baecke Habitual Physical Activity Questionnaire (BHPAQ) is a self-administered, self-report tool designed to evaluate physical activity over the past 12 months. It comprises 16 items, divided into three domains: occupational physical activity (items 1–8), sports-related physical activity during free time (items 9–12), and leisure-time physical activity not related to sports (items 13–16). Responses are rated on a five-point Likert scale (1–5). The occupational domain score is determined by adding the responses for all items in the domain and dividing the total by 8, with a specific adjustment for item 2 (subtracting 6 from the response value). For the sports domain, the score is calculated by summing the responses and dividing by 4. Similarly, the leisure domain score is obtained by summing the responses and dividing by 4, with an adjustment for item 13 (subtracting 6 from the response value). Each domain’s final score ranges from 1 to 5, where a higher score indicates a greater level of physical activity.

Handgrip strength was measured using a Camry digital dynamometer, model EH101 (Guangdong, China). The test was performed duplicate, with the individuals sitting in a chair without arm rests, the shoulder slightly adducted and the elbow of the dominant arm (the arm that a person uses more frequently and with greater skill or strength) flexed at 90° and with the forearm and wrist in a neutral position. The participants were instructed to squeeze the dynamometer as hard as possible twice with a 1-min interval between each attempt. The value of the peak force (the maximum value in kg. f applied on the dynamometer) was recorded. EWGSOP2 (Cruz-Jentoft et al. (2019) provide recommendations for cut-off points for grip strength: <27 kg. f for men; <16 kg. f for women.

### 2.5 Statistical analysis

Initially, the Shapiro-Wilk test was used to analyze data distribution. Information on central tendency and dispersion of the data (descriptive characteristics and BIA data) were presented as mean and standard deviation. Chi-square test was performed to compare the frequencies between genders according to the groups/categories. Comparisons of the BIA variables were performed using confidence ellipses, and statistical analysis by the Hotelling’s T^2^ test, with the presentation of the Mahalanobis’ D distance and its respective significance value. For all statistical analyses, significance were set at 0.05. Data was recorded and analyzed using SPSS statistical packages for Windows, version 29.0 (SPSS Inc., Chicago, IL, USA), and confidence ellipses using the BIVA Software 2002 (Department of Medical and Surgical Sciences, University of Padova, Italy).

## 3 Results

A total of 187 subjects (143 females and 44 males) were enrolled in the present study and underwent body composition and functionality assessments. Participants were then classified according to sex (men; women). Men reportedly had higher significant values for age, body weight, height, waist circumference and lean soft tissue (LST). Morphological and anthropometric characteristics of the participants are presented in Table 1.

**TABLE 1:** Descriptive characteristics (women = 143; men = 44).

Variable	Older women		Older men		*p*
	Mean ± SD	Minimum - Maximum	Mean ± SD	Minimum - Maximum	
Age (years)	70.5 ± 6.8	58.2–87.3	74.9 ± 7.8	60.9–93.0	0.000
Body weight (kg)	68.3 ± 13.1	43.8–121.9	75.1 ± 13.1	52.8–113.9	0.003
Height (cm)	155.0 ± 6.6	141.4–178	166.7 ± 5.6	154.0–179.6	0.000
BMI, (kg/m^2^)	28.4 ± 5.1	18.4–45.9	26.9 ± 4.1	18.3–39.0	0.086
Relaxed arm circ., (cm)	30.5 ± 3.7	22.0–42.0	29.5 ± 3.4	22.5–40.1	0.132
Waist circ., (cm)	93.7 ± 14.0	68.0–145.0	99.2 ± 13.2	66.5–126.4	0.022
Calf circ., (cm)	35.8 ± 3.4	28.7–44.0	35.2 ± 3.1	29.0–42.7	0.246
Occupational score	1.3 ± 1.5	0.0–3.8	1.4 ± 1.4	0.0–3.6	0.659
Exercise and leisure score	1.9 ± 0.5	0.8–4.0	1.8 ± 0.4	1.0–2.8	0.249
Locomotion score	2.0 ± 0.6	1.0–3.5	2.0 ± 0.6	1.0–3.0	0.711
Total physical activity score	5.1 ± 1.7	2.3–8.8	5.1 ± 1.6	2.8–8.6	0.949
Hand grip strength (kg)	21.3 ± 4.3	12.3–34.2	31.2 ± 7.7	15.5–50.7	0.000

Notes: Values are presented as mean ± SD; kg, kilograms; cm, centimeters; BMI, body mass index; kg/m^2^; bodyweight in kilograms over height in meters squared, p = significance level referring to Student’s t-test for independent samples.

In the analysis of the frequencies of women and men according to the different groups analyzed, a different and statistically significant distribution was verified only for the age group variables, with more men in the age groups over 70 years (Table 2).

**TABLE 2:** Frequency of women and men According to analyzed groups (women = 143; men = 44).

Variable	Categories	Older women		Older men		*p*
		N	%	N	%	
Age group	60–69 years	77	53,8	12	27,3	0,006
	70–79 years	50	35,0	22	50,0	
	80 years or older	16	11,2	10	22,7	
Physical activity level	Normal level of physical activity	102	71,3	31	70,5	0,911
	Low level of physical activity	41	28,7	13	29,5	
Handgrip strength	Normal handgrip strength	85	59,4	23	52,3	0,400
	Low handgrip strength	58	40,6	21	47,7	

Notes: Values are presented as n (%). N, number of participants; *p* = significance level for chi-square test.

In Table 3, when separated into groups, R and Xc variables were different between the classic and specific proposals. Higher values were reported for the classic proposal, in all groups, with a greater number of differences for women.

**TABLE 3:** Comparison of BIA parameters (R, Xc and PhA) according to classic and specific proposal, gender, level of physical activity and handgrip strength (women = 143; men = 44).

	Older women					Older men				
	R/H	Xc/H	R.sp	Xc.sp	PhA	R/H	Xc/H	R.sp	Xc.sp	PhA
Normal physical activity level										
Mean	580,1	52,8	488,9	44,6	5,2	489,5	45,5	416,6	38,9	5,3
SD	68,8	9,1	78,2	9,4	0,7	68,7	9,4	64,6	9,2	0,9
Low level of physical activity										
Mean	572,3	46,5	544,8	44,6	4,7	512,9	47,8	410,0	38,4	5,3
SD	75,1	7,6	82,8	9,9	0,8	40,8	6,6	56,2	8,3	0,7
Normal handgrip strength										
Mean	576,7	51,9	505,7	45,6	5,2	470,7	46,6	419,3	41,4	5,6
SD	66,1	9,3	88,7	10,3	0,8	56,8	10,2	57,5	8,7	0,9
Low handgrip strength										
Mean	579,5	49,8	503,8	43,2	4,9	524,6	45,6	409,5	35,9	5,0
SD	76,9	8,9	75,3	8,2	0,7	56,5	6,7	66,9	8,3	0,6

Notes: Values are presented as mean and SD: standard deviation; R/H = resistance in the classic model; Xc/H = reactance in the classical model; R. sp = resistance in the specific model; Xc. sp = reactance in the specific model; PhA = phase angle; SD, standard deviation.

**FIGURE 1:**
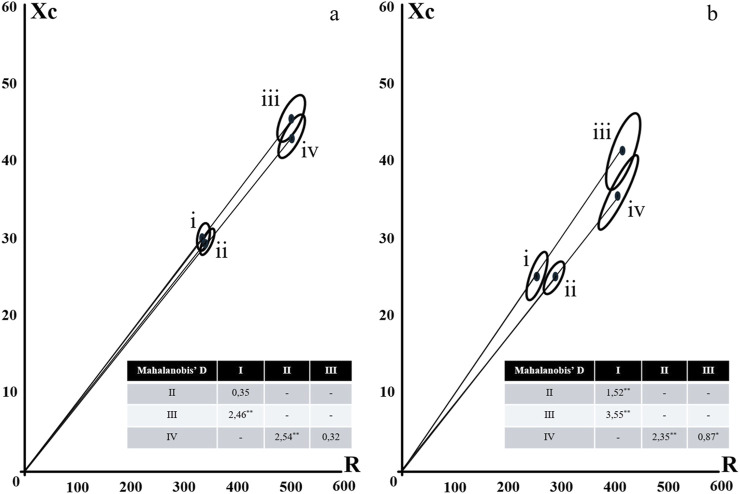
Confidence Ellipses for Classic (i and ii) and Specific (iii and iv) BIVA according to gender [**(a)** female; **(b)** male] and level of physical activity (i and iii = normal and high level; ii and iv = low level). Notes: * = Mahalanobi’s D distance significance at p < 0.05; ** = Mahalanobi’s D distance significance at p < 0.001.

**FIGURE 2:**
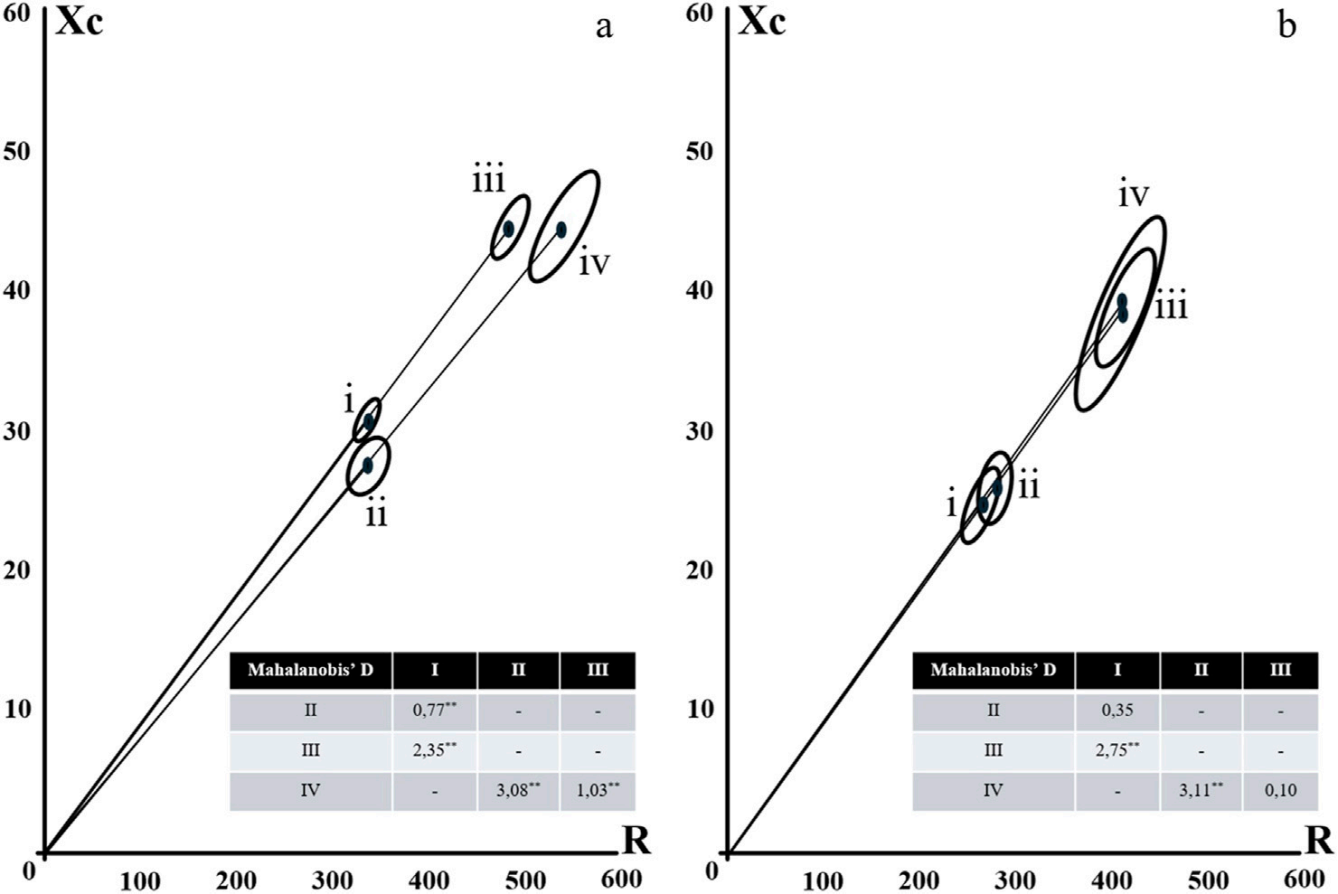
Confidence Ellipses for Classic (i and ii) and Specific (iii and iv) BIVA according to gender [**(a)** female; **(b)** male] and strength (i and iii = normal strength; ii and iv = low strength). Notes: * = Mahalanobi’s D distance significance at p < 0.05; ** = Mahalanobi’s D distance significance at p < 0.001.

In the vector analyses, based on the confidence ellipses, between male and female (Figures 1, 2), despite the higher Xc values verified for women, the PhA was higher for men, aided by the lower R values, causing that the groups were statistically different, without the overlapping of the ellipses. The behavior of the two evaluation proposals was similar.

No differences were reported for level of physical activity between men in the most active group when compared to men in the least active group for both the classic and specific proposals (Figure 1). However, women in the most active group presented with greater PhA and differences of the ellipses, for both the classic and specific BIVA proposals. Differences between the classic proposals are due to the higher value of Xc, while in the specific proposal, due to the higher values of R.

In the analysis of the vectors for strength comparison, the patterns were the opposite, where the stronger women did not show differences compared to the weaker women, either for the classical or for the specific analysis, while for the men, the stronger ones had ellipses higher up, with higher phase angles, due to the lower R values ​​in the classical analysis, and higher Xc values ​​in the specific analysis (Figure 2).

## 4 Discussion

The objective of the present study was to analyze the differences in the values of R, Xc and PhA between the two proposals: classic and specific BIVA according to levels of physical activity and sex-specific factors. No differences for PhA were anticipated as the same adjustments were made for R and Xc, when converting values to the specific model. Therefore, the PhA values are maintained.

For the category of obese people, for both male and female, both variables (R and Xc), statistically significant differences were reported by the non-overlapping confidence intervals (95% CI) of the mean values of R and Xc. These findings were verified for all the other analyzed groups, according to sex. Values for the classic model were 4%–30% higher, with the same pattern for R and Xc.

In the case of obesity, women had an average value of 8% higher for R and Xc for the specific models, and men had an average value of 5%. This situation occurs precisely because of the biophysical principle of BIA considering the direct relationship between the cross-sectional area of the segment and the values of R (Buffa et al., 2013).

In the BIVA analyses, the lean and muscular components were of high importance in the analysis of functionality and sarcopenia in older adults. The vectors of the classic model were slightly longer when compared to the vectors of the specific models, in addition, a greater difference was observed between males and females for the classic model. The results are consistent with the theoretical expectations of the BIVA, since the sarcopenic individuals classified by the BIA were located in the region of the graph corresponding to thin individuals, with less cell mass, in fact. In general, a low PhA is related to a low body cell mass (Piccoli et al., 1994).

Our findings are comparable to the previous literature with regard to the behavior of muscle components. Lower PhA values and a high R/H, with lower Xc/H values were observed in cachectic individuals (Castillo-Martínez et al., 2012), as well as the same displacement pattern was verified for individuals with lower muscle strength (evaluated by handgrip strength, as in the present study), highly related to muscle mass (Norman et al., 2009). Both authors mentioned interpreted this displacement of vectors between the groups with greater and lesser strength as indicative of low muscle function. The use of BIVA is suggested instead of handgrip strength tests when patients are unable to properly perform the test. Similarly, the peculiar bioelectric pattern found in sarcopenic individuals may be due to the decline in muscle function (Norman et al., 2009).

In the present study, we used the appendicular LST index to classify subjects with low muscle mass and the sarcopenia diagnostic model proposed by the European consensus (Cruz-Jentoft et al., 2019; Marini et al., 2013) found a significant relationship between the PhA and LST index and muscle mass, confirmed the expectations of Piccoli et al. (1994). Similar results were noticed by Kyle et al. (2003) who found a positive relationship between reactance and LST. Mean difference for the relative amount of fat mass for specific BIVA, in both sarcopenic and non-sarcopenic individuals, were not evident, when compared to the classic model. However, BIVA presents to be more adequate in body composition analyses, when evaluating fat components, than anthropometric proposals, such as BMI and isolated circumferences (Marini et al., 2013).

The use of vector analysis, rather than predictive equations, accounts for the electrical properties of tissues and is sensitive to changes in cell mass and hydration. This is achieved by utilizing raw values of R and Xc normalized by height, regardless of whether classical or specific approaches are used (Piccoli et al., 1994; Norman et al., 2009). Consequently, BIVA has been shown to more accurately assess total body fat mass and fat-free mass, as well as skeletal muscle mass, compared to predictive equations. In both classical and specific BIVA models, variables such as gender and physical activity level aligned with existing standards, yielding positive outcomes for cellular health, cellular integrity, and body composition. The results within the groups highlighted variables related to muscular function, such as faster gait speeds, greater improvements (>8 in SPPB test) in physical performance, and higher levels of independence in performing activities of daily living.

This situation validates the equipment for evaluating not only the morphological components, but also the functional components, remembering that in some situations, such as walking speed, there was a slight overlap of the ellipses in both groups (male and female), but indicating a tendency for the best in functional condition to present better BIA values.

There is limited research surrounding functional variables, such as walking speed, mobility score, activities of daily living and frailty, focusing on the use, comparison and correlation of the classic or specific model among different ages and groups based on levels of physical activity as was done in the present study.

There were potential limitations in our cross-sectional study such as a lack of gender diversity with most participants being female. A larger sample size and power may allow further analysis.

The classic and specific proposals for the analysis of bioelectrical impedance vectors, despite being calculated using different methodologies, show similar patterns in the positioning of vectors of confidence ellipses in the graphs, especially when groups of older adults are compared according to functionality variables. Additional studies are needed with different tests and evaluations, to confirm the validity of the models, however, the use of BIVA analyses, regardless of the proposal used, appears to follow a logical explanation for older adults.

## Data Availability

The original contributions presented in the study are included in the article/supplementary material, further inquiries can be directed to the corresponding author.
